# Seroprevalence and associated behavioral factors of *Toxoplasma gondii* infection among pregnant women in Pokhara Valley, Nepal

**DOI:** 10.1186/s41182-025-00803-8

**Published:** 2025-09-30

**Authors:** Mamata Thapa, Tulsi Ram Gompo, Tatsuki Sugi, Masahito Asada, Hiroaki Arima, Kishor Pandey

**Affiliations:** 1 Central Department of Zoology, Institute of Science and Technology, Tribhuvan University, Kirtipur, Kathmandu, Nepal; 2 Central Veterinary Laboratory, Kathmandu, Nepal; 3 Melbourne Veterinary School, University of Melbourne, Parkville, Melbourne, Australia VIC 3010; 4 Division of Collaboration and Education, International Institute for Zoonosis Control, Hokkaido University, Sapporo, 001-0020 Japan; 5 National Research Center for Protozoan Diseases, Obihiro University of Agriculture and Veterinary Medicine, Obhiro, 080-8555 Japan; 6 Department of International Health and Medical Anthropology, Institute of Tropical Medicine, Nagasaki University, 1-12-4 Sakamoto, Nagasaki, 852-8523 Japan

**Keywords:** *Toxoplasma gondii*, RDT, ELISA, Seroprevalence, Pregnancy, Risk factors, Nepal

## Abstract

**Background:**

*Toxoplasma gondii* is a protozoan parasite that can cause congenital infections with serious health implications. In pregnant women, *T. gondii* infection poses significant risks, including miscarriage, stillbirth, and congenital abnormalities in the fetus. The burden of toxoplasmosis is often underrecognized in many developing countries, including Nepal, where awareness and routine screening are limited. This study was aimed at determining the prevalence of *T. gondii* infection among pregnant women in Pokhara Valley, Nepal, and to identify behavioral risk factors associated with the transmission of this parasite.

**Methods:**

A cross-sectional study was conducted at Gandaki Medical College Teaching Hospital, Pokhara Valley, Nepal, from October 2024 to January 2025. A total of 257 serum samples were collected, 91 of which were randomly selected and tested for anti-*T. gondii* IgG/IgM antibodies using a rapid diagnostic test (RDT) and enzyme-linked immunosorbent assay (ELISA). Sociodemographic and behavioral data were collected using structured questionnaires. Statistical analyses included Fisher’s exact test to assess associations and Cohen’s kappa coefficient to evaluate the consistency between the two diagnostic methods.

**Results:**

The RDT detected IgG antibodies in 19.78% (18/91) of the participants, whereas the ELISA identified 38.46% (35/91) of the participants as seropositive. No IgM-positive cases were detected by either method. Compared with the ELISA, the RDT exhibited low sensitivity (34.3%) but high specificity (89.3%), with fair consistency (kappa = 0.26). Cat ownership showed a borderline significant association with seropositivity (OR = 3.79, *p* = 0.05). Notably, none of the participants demonstrated any knowledge of toxoplasmosis (0%).

**Conclusions:**

The findings reveal a significant public health concern. The relatively high seroprevalence of *T. gondii*, combined with a lack of awareness and identifiable risk factors, underscores the urgent need for educational interventions and prenatal screening programs to reduce the risk of congenital toxoplasmosis in Nepal.

## Background

*Toxoplasma gondii* (*T. gondii*) is a single-celled, obligate intracellular parasite that represents a single species within the genus *Toxoplasma* [1]. Cats and felids (e.g., tigers and leopards) are the definitive hosts in which the parasite undergoes sexual reproduction and forms oocysts in the small intestine. Warm-blooded animals, including goats, sheep, rodents, and humans, serve as intermediate hosts in which tissue cysts develop. *T. gondii* progresses through three main stages: tachyzoites, bradyzoites, and sporozoites [2]. The tachyzoite stage is associated with acute infection and rapid replication of the parasite in intermediate hosts, whereas bradyzoites form tissue cysts during chronic infection. In definitive hosts, the parasite undergoes sexual reproduction, resulting in the shedding of unsporulated oocysts. These oocysts sporulate in the environment, where they develop into sporozoites, the infectious form for intermediate hosts [3]. Intermediate hosts, including humans, can become infected through various routes: ingestion of water, vegetables, or fruits contaminated with sporulated oocysts from cat feces [4]; consumption of raw or uncooked meat containing tissue cysts; or blood transfusions and organ transplants [5].

Toxoplasmosis is an important parasitic disease that infects approximately 30–50% of the global human population [6]. *T. gondii* infection is typically asymptomatic in immunocompetent individuals. However, it causes serious complications in pregnant women, individuals with weakened immune systems and those with HIV/AIDS [7]. During pregnancy, the infection can be transmitted congenitally to the fetus [8], potentially resulting in hydrocephalus, microcephaly, calcifications within the brain, retinochoroiditis, strabismus, visual impairment, epilepsy, delayed development, intellectual disability, petechiae due to low platelet count, and anemia [9]. Acute toxoplasmosis is usually acquired through the consumption of contaminated food or environmental exposure, leading to systemic spread and symptoms depending on the immune response. In contrast, chronic infection involves long-term persistence of tissue cysts, which may occasionally trigger inflammation, especially in sensitive tissues such as the eye or brain [10]. The risk of fetal infection increases with gestational age: 15% at the 13th week of pregnancy, 44% at the 26th week, and 71% at the 36th week [5]. While early pregnancy infections are less likely to be transmitted, they tend to cause more severe fetal harm. Conversely, infections acquired late in pregnancy may appear mild at birth but can lead to eye and neurological problems later [9].

Several behavioral and environmental factors, including dietary habits, hygiene practices, and environmental conditions, can influence the risk of *T. gondii* infection. Previous studies have identified several risk factors for *T. gondii* infection, including living in rural areas, working with soil, consuming raw or undercooked meat, drinking unpasteurized milk, having a history of miscarriage and having cats in the home, using unimproved water sources, and having a lower socioeconomic status [11–14]. Nepal's first confirmed case of congenital toxoplasmosis was in a 53-day-old infant [15]. There are very few studies about toxoplasmosis in Nepal [16–22]. Most of these studies focused on antibody detection in animals and immunocompromised humans [15, 17, 19, 20]. According to the Nepali government, toxoplasmosis is classified among the country’s top ten zoonotic diseases [23]. In Nepal, the epidemiological situation of *T. gondii* infection among young women and pregnant women is not well understood, and its prevalence remains unknown in many regions. Furthermore, *Toxoplasma* screening is not included in routine prenatal care. Therefore, investigating the infection status among pregnant women is important for understanding the risk of congenital toxoplasmosis and identifying behavioral factors associated with transmission. While several studies in Nepal have reported bacterial contamination in dairy and meat products [24–26], research focusing on protozoan parasites such as *T. gondii* is extremely limited. In particular, very few studies have assessed *Toxoplasma* infection among pregnant women. In some parts of Nepal, traditional dietary practices, such as the consumption of unpasteurized milk and raw meat, still persist [24, 27], which may increase the risk of infection. For example, the traditional Newari dish "kachela" is made from raw buffalo meat. Even frozen buffalo meat has been reported to carry a risk of *T. gondii* infection [28]. A recent meta-analysis revealed that individuals who consume raw or undercooked meat have a 1.2–1.3 times greater risk and 1.7–3.0 times greater odds of *T. gondii* infection than do those who consume thoroughly cooked meat [29]. Therefore, the present study was aimed at determining the seroprevalence of *T. gondii* infection and identifying the associated behavioral risk factors among pregnant women in Pokhara Valley, an urban area in western Nepal.

## Methods

### Study site and sample collection

**Fig. 1:**
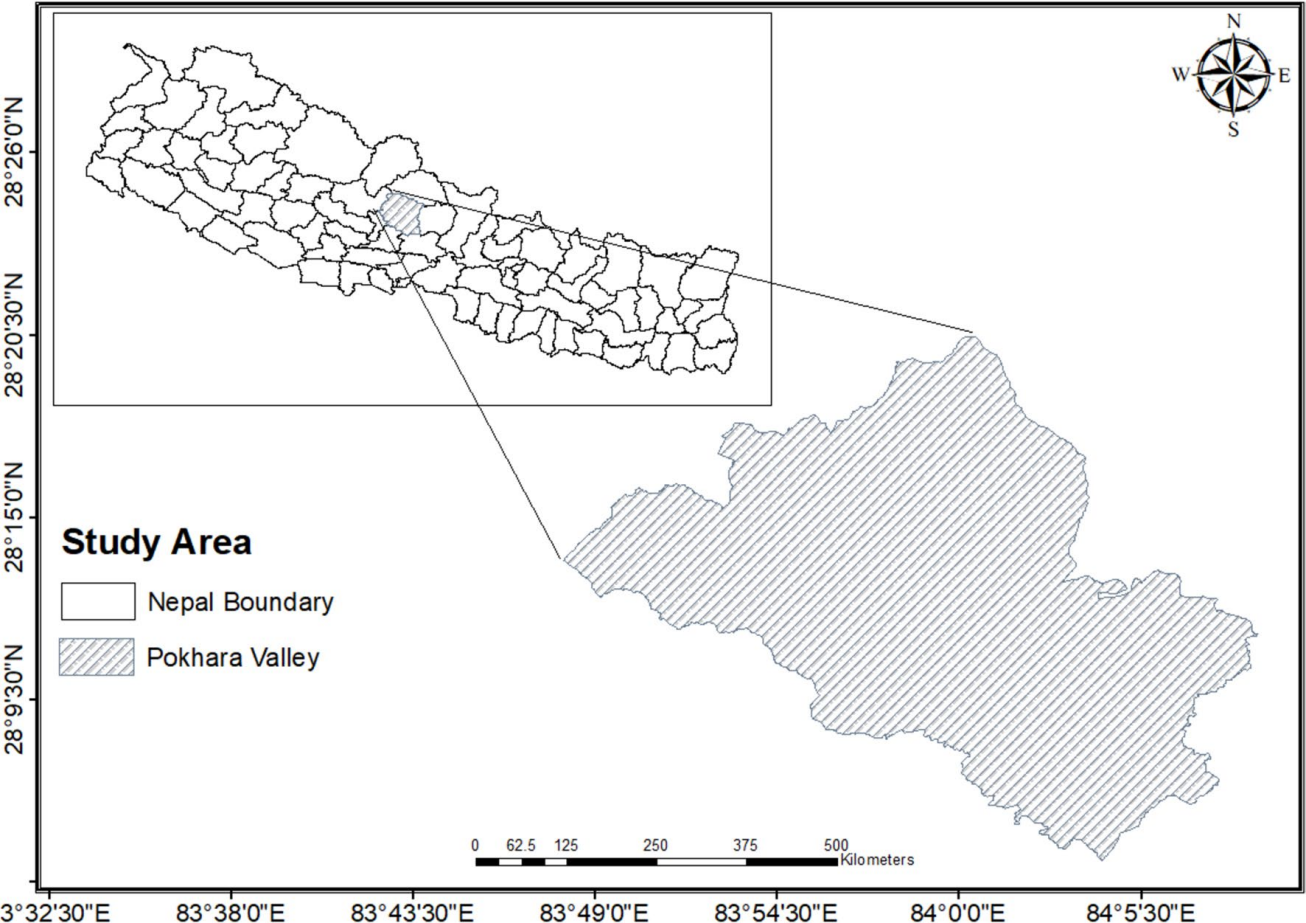
Map of Nepal showing the study site. This map illustrates the geographic location of Pokhara Valley, where the participants were recruited and data collection took place for the present study

This study was conducted at Gandaki Medical Teaching Hospital (GMTH), which is located in Pokhara Valley, Kaski district, Nepal (Fig. 1). Data were collected through direct interviews using a pretested oral questionnaire after informed consent was obtained from each respondent. Time-based convenience sampling was used to select pregnant women who visited GMTH for routine health checkups and provided blood samples. Samples were collected between October 2024 and January 2025. After collection, the serum samples were transported and stored at the Tribhuvan University laboratory under appropriate conditions for approximately 3 months. Pregnant women undergo regular blood tests to monitor their pregnancy status. In this study, we used 3 ml from each of these blood samples to measure antibodies against *T. gondii*. Hemoglobin and platelet count data were obtained from hospital laboratory records, as the participants were already undergoing regular clinical evaluations. These values were measured using an automated hematology analyzer (Sysmex Coulter Counter) to determine the hemoglobin concentration and platelet count. Rapid diagnostic tests (RDTs) were conducted at the hospital to detect *Toxoplasma gondii*-specific IgM and IgG antibodies, and the remaining samples were stored at -20 °C. All the samples were transported to the Central Department of Zoology, Tribhuvan University, Kirtipur, where they were maintained at -20 °C until ELISA analysis.

## Questionnaire

A structured questionnaire was used to interview the pregnant women. The collected information contained three broad groups—sociodemographic profiles, including age, level of education, occupational status, gestation period, and parity; knowledge questions related to *Toxoplasma* infection during pregnancy; and risk factor-related questions associated with toxoplasmosis. The risk factors included patient age group, educational status, occupation, source of drinking water, habit of eating raw meat, and owning cats at home.

## Detection of anti-*Toxoplasma* antibodies using RDT and ELISA

We used Toxo IgG/IgM RDT kits (Neo-nostics, Jiangsu, China) to detect the antibodies against *T. gondii* in accordance with the manufacturer’s instructions. The kit components were heated to room temperature prior to use. Ten µL of serum or whole blood was added to the cassette, followed by the addition of two drops of buffer. After 15 min of incubation, the results were interpreted as follows: two lines indicated positive results, one control line indicated negative results, and no control line rendered the test invalid. Additionally, the anti-*T. gondii* antibodies in the serum samples were tested using anti-*T. gondii* IgG and IgM Human ELISA kits (Abcam, MA, USA). Both the IgG and IgM antibodies were measured quantitatively using ELISA, with IgG concentrations being expressed in IU/mL and IgM interpreted on the basis of OD values relative to the cutoff. The optical density (OD) values for the ELISA tests were read using a BioTek Epoch 2 microplate reader. The results were interpreted according to the manufacturer’s instructions. For IgG, a sample was considered reactive (positive) if the antibody concentration was greater than 35 IU/mL, inconclusive if it was between 30 and 35 IU/mL, and nonreactive (negative) if it was less than 30 IU/mL. For IgM, a sample was considered positive if its optical density (OD) value exceeded the cutoff by more than 10% (i.e., > 110% of the cutoff value). Samples with OD values within ± 10% of the cutoff were considered inconclusive (gray zone), whereas those with OD values greater than 10% below the cutoff were considered negative. A positive result was visually indicated by a color change in the ELISA well, also referred to as a positive well. The RDT results were compared with the ELISA results using classification matrices, with ELISA serving as the reference standard. The ELISA method used here had reported sensitivity (96.6%) and specificity (98.2%) levels [30], providing a benchmark for evaluating the RDT performance.

## Statistical analysis

The raw data from the questionnaire and test results were combined to create complete metadata. All the data analyses were conducted using R version 4.4.3.2 (2021-11-01). Concordance between RDT and ELISA was assessed by calculating Cohen’s kappa coefficient (k) with interpretation based on standard kappa cutoff values [31]. The ELISA results were used to classify participants as infected or uninfected, and these classifications were analyzed alongside the questionnaire data to identify significant risk factors associated with infection. The relationships between variables were examined using Fisher’s exact test, with p values of less than 0.05 considered statistically significant. Additionally, the odd ratios (ORs) and 95% confidence intervals (CIs) were calculated to determine the strength of the associations.

## Ethics approval

Ethical approval was obtained from the Institutional Review Committee of the Institute of Science and Technology, Tribhuvan University (210/081/081), and the Institutional Review Committee of Gandaki Medical College (440/080/081), and written informed consent was obtained from all the participants during the interviews and sample collection.

## Results

### Seroprevalence of *Toxoplasma* infection

A total of 257 participants were enrolled in this study. Among these, 91 were randomly selected for serological testing using RDT and ELISA to ensure the effective use of research resources, minimize selection bias in prevalence estimation, and allow for appropriate generalization to the broader population. Geographically, 91.21% (83/91) of the participants were from the Kaski district, whereas 8.79% (8/91) were from neighboring districts. Among the 91 samples tested by RDT, 19.78% (18/91) were positive for IgG antibodies. None of the samples tested positive (0/91) for IgM antibodies. All the samples were retested using IgG and IgM ELISA. The ELISA results revealed that 38.46% (35/91) of the samples were IgG positive, whereas no IgM positivity (0/91) was detected among the tested samples. Among these, 6 (6.59%) that were RDT positive were ELISA negative, 23 (25.27%) were ELISA positive but RDT negative, and 12 (13.18%) were positive by both RDT and ELISA (Table 1).

**Table 1:** Seroprevalence of *Toxoplasma gondii*

Test		ELISA		Total
		Positive	Negative	
RDT	Positive	12 (13.18%)	6 (6.59%)	18 (19.78%)
	Negative	23 (25.27%)	50 (54.95%)	73 (80.22%)
Total		35 (38.46%)	56 (61.55%)	91 (100.00%)

This table summarizes the IgG antibody test results obtained using RDT and ELISA. No IgM-positive cases were detected among the test samples. The data in each cell are presented as the *n* (%)

## Diagnostic parameters of RDT versus ELISA

The comparison revealed that the RDT had a sensitivity of 34.3% and a specificity of 89.3%. The positive predictive value of the RDT was 66.7%, and the negative predictive value was 68.9%. However, the overall accuracy was only 68.1%. The Cohen’s kappa estimate of 0.26 indicated only fair agreement with the ELISA results. The area under the curve (AUC) value of 0.62 from the receiver operating curve (ROC) indicates fair diagnostic ability (Table 2).

**Table 2:** Diagnostic parameters of RDT versus ELISA

Diagnostic	Sensitivity	Specificity	Accuracy	PPV	NPV	Cohen's kappa	AUC
RDT-IgG	34.3%	89.3%	68.1%	66.7%	68.9%	26.0%	62.0%

This table presents the diagnostic parameters of RDT-IgG using ELISA as the reference standard. The values shown here include the sensitivity, specificity, accuracy, positive predictive value (PPV), negative predictive value (NPV), Cohen’s kappa coefficient, and the area under the receiver operating characteristic curve (AUC)

## Hematological parameters

The associations between hemoglobin levels, platelet counts, and *Toxoplasma* IgG positivity are shown in Table 3. The criteria for classifying “low” and “normal” hemoglobin levels and platelet counts were based on the standard clinical reference range (hemoglobin level: 12–16 g/dl; platelet count: 150,000–450,000 cells/mm3). No statistically significant associations were found for any of the variables we examined. Hemoglobin levels showed a borderline statistical association with IgG positivity (OR = 0.43, 95% CI: 0.16–1.15, *p* = 0.08), whereas the platelet count was not significantly different (OR = 0.80, 95% CI: 0.01–15.84, *p* = 1.00), with a wide confidence interval indicating uncertainty in the estimate.

**Table 3:** Effects of *Toxoplasma gondii* infection on hematological parameters

Variable	Category	IgG		Odd Ratio (95% CI)		*p* value
		Positive	Negative			
Hemoglobin(g/dL)	Low	10	27	0.43	(0.16–1.15)	0.08
	Normal	25	29			
Platelets (cells/mm^3^)	Low	1	2	0.8	(0.01–15.84)	1.00
	Normal	34	54			

This table presents the associations between hematological parameters—the red blood cell count and platelet count—and IgG seropositivity. The number of individuals in each IgG-positive and IgG-negative group is shown as *n*

## Sociodemographic profiles and risk factors associated with *T. gondii* seropositivity

Most participants were aged 18–29 years (74.72%, 68/91) and had secondary education (51.65%, 47/91) (Table 4). Most participants were in their first pregnancy (63.75%, 58/91), followed by those with second pregnancies (32.96%, 30/91) and third pregnancies (3.29%, 3/91). The majority were housewives (72.52%, 66/91), and most were in their third trimester of pregnancy (45.05%, 41/91).

**Table 4:** Demographic characteristics of the study population

Variable	Category	Frequency (*n* = 91)	Percentage
Age group	18–29	68	74.72
	30–39	23	25.28
Education	Primary	11	12.08
	Secondary	47	51.65
	Tertiary	33	36.27
Occupation	Housewife	66	72.52
	Other	25	27.48
Gestational age	First trimester	26	28.58
	Second trimester	24	26.37
	Third trimester	41	45.05
No. of pregnancy (parity)	One	58	63.75
	Two	30	32.96
	Three	3	3.29

This table shows the number and percentage of participants in each category based on their demographic characteristics

## Risk factors associated with *Toxoplasma* seroprevalence in the present study

There were no significant associations with age, education, occupation, drinking water source, raw meat consumption, miscarriage history, eating habits, or awareness (Table 5). Although raw meat consumption did not have a statistically significant association (p = 0.56), the odds ratio was relatively high (OR = 3.33), suggesting a possible increased risk. However, the small number of participants in this group may have limited the statistical power, and the results should be interpreted with caution. Similarly, cat ownership had a greater odds ratio (OR = 3.85), suggesting a possible risk factor (*p* = 0.05) that was close to statistical significance, indicating a potential association. An OR = 1 indicates no association; an OR > 1 suggests that exposure may be a risk factor for the disease; and an OR < 1 suggests that exposure may be protective against the disease [32].

**Table 5:** Risk factors associated with *Toxoplasma* seroprevalence in the present study

Risk factor	Category	IgG		Odd Ratio (95% CI)						p value
		Positive	Negative							
Age group (years)	18–29	27	41	1.23	(0.46–3.10)					0.81
	30–39	8	15							
Education	Basic	4	7	0.90	(0.24–3.42)					1.00
	Higher	31	49							
Occupation	Housewife	25	41	0.91	(0.35–2.34)					1.00
	Other	10	15							
Gestational age	First trimester	9	17	Ref						0.68
	Second trimester	8	16	0.94	(0.34–2.61)					
	Third trimester	18	23	1.47	(0.29–3.76)					
No. of pregnancy (parity)	One	24	34	Ref						0.38
	Two	9	21	0.61	(0.24–1.53)					
	Three	2	1	2.83	(0.25–32.27)					
Drinking water	Treated	16	28	0.84	(0.36–1.96)					0.83
	Untreated	19	28							
Raw meat-eating habit	Yes	2	1	3.33	(0.29–38.20)					0.56
	No	33	55							
Owning cats	Yes	8	4	3.85	(1.06–13.95)					0.05
	No	27	52							
Miscarriage	Yes	0	2	0.00	(0.00–8.53)					0.52
	No	35	54							
Eating habit	Veg	1	6	0.25	(0.21–2.12)					0.24
	Nonveg	34	50							
Awareness	Yes	0	2	0.00	(0.00–8.53)					0.52
	No	35	54							

This table shows the comparison of the IgG seropositivity rates across categories on the basis of the participants’ demographic characteristics and lifestyle factors. *P* values were derived from Fisher’s exact test

## Discussion

This study assessed the seroprevalence of *T. gondii* infection and its associated risk factors in pregnant women receiving antenatal care at GMRH in Pokhara Valley according to both commercial RDT kits and ELISA. Toxoplasmosis during pregnancy is typically diagnosed through serological testing for IgG and IgM antibodies. In our study, the ELISA detected a higher IgG seroprevalence (38.46%) than did the RDT (19.78%). This finding is consistent with previous studies in Ghana, which reported an IgG seroprevalence of 57.3% by ELISA compared with 21.5% by RDT, further supporting the greater reliability of ELISA for diagnosing *T. gondii* infection [33]. The presence of IgG (38.46%) and the absence of IgM antibodies in our findings are slightly lower than those reported in a previous study conducted in Pokhara, which reported IgG at 11.54% and IgM at 1.92% based on chemiluminescence immunoassay results [34]. The sensitivity (15.8%) and specificity (95%) of the RDT observed in a previous study from Egypt were consistent with our findings [35]. However, a contrasting study reported a higher sensitivity (88%) and lower specificity (89.29%) for ELISA in detecting IgG antibodies for the diagnosis of toxoplasmosis [36]. In our study, the diagnostic performance of RDT-IgG showed fair consistency with that of ELISA, with a kappa value of 0.26 and an AUC of 0.62. These results are comparable to those of a study from Ghana, which reported a kappa of 0.20 and an AUC of 0.61, suggesting that the RDT has poor to fair discriminatory ability in detecting *T. gondii* antibodies. The same study reported a prevalence of 21.5% using RDT and 57.3% using ELISA [33]. The variation in performance could be attributed to differences in sensitivity and specificity among RDT kits from different manufacturers. Additionally, while the observed IgG seroprevalence aligns with that reported in other studies, the cross-sectional design of our study does not allow for an assessment of consistency over time. No IgM positivity was detected, likely because the participants were asymptomatic pregnant women attending routine checkups.

Similarly, a study from Bannu District, Pakistan, reported an overall prevalence of only 1.32% [37], which was markedly lower than the prevalence reported in our study. In Alexandria, Egypt, a study using both RDT and ELISA reported prevalence rates of 11.3% and 57.9%, respectively, again demonstrating the limited diagnostic performance of RDT compared with the more reliable ELISA method [35]. These differences in prevalence and diagnostic performance may be attributed to variations in diagnostic tools, dietary habits, environmental conditions, and cultural factors influencing exposure to *T. gondii*.

The relatively consistent IgG prevalence across previous studies suggests a stable endemicity of *T. gondii* in this region. However, the absence of IgM positivity in our study may reflect differences in sample sizes, demographic characteristics, or diagnostic methods. When the prevalence of *T. gondii* across geographical regions is compared, it shows significant variability. Our IgG seropositivity rate of 38.46% is substantially higher than the 18.82% IgM seropositivity reported among women of childbearing age in Kathmandu [38]. In Sunsari District, Nepal, 150 human samples were tested using the Toxo IgG/IgM Combo Rapid Test, with a seroprevalence of 12.67%. Toxoplasmosis was highly significantly associated with abortion, as 58.33% of seropositive individuals had a history of abortion [39]. This is the first report from Nepal to assess *T. gondii* infection among 45 HIV/AIDS patients at the National Public Health Laboratory, Teku, Kathmandu. IgG antibodies were detected in 33.3% (15/45) of the patients, while none tested positive for IgM [21]. For example, a study from Gampaha District of Sri Lanka that used an RDT reported IgG and IgM seroprevalence rates of 12.3% and 0%, respectively [40].

Furthermore, consistent with findings from the Ashanti region of Ghana, our study revealed no significant association between *T. gondii* and hemoglobin levels in pregnant women [41]. These findings suggest that toxoplasmosis may not have a direct effect on maternal hemoglobin status, although further research is warranted to explore this relationship in greater detail.

Cat ownership was a potential risk factor for *T. gondii* seropositivity in our study. This finding aligns with research from Ghana, where cat contact and lower educational attainment were identified as major risk factors for *T. gondii* infection [33]. In the Nepali context, cats are commonly kept as pets without adequate care and are often allowed to roam freely and hunt rodents, increasing the risk of environmental contamination with oocysts. These sociocultural practices likely contribute to *T. gondii* transmission and underscore the need for public awareness programs promoting responsible pet ownership, including proper feeding, regular deworming, and safe disposal of cat feces, to help mitigate the risk of infection [42].

Several studies have documented a strong link between raw meat consumption and *T. gondii* infection. For example, one study identified a history of consuming raw meat as a significant risk factor for toxoplasmosis [43]. Similarly, research conducted in southwestern Iran revealed that the consumption of undercooked meat was significantly associated with increased IgG seroprevalence (*p* = 0.007) [44]. However, in our study, while raw meat consumption had a high odds ratio (OR = 3.29), the association was not statistically significant (p = 0.56).

The relationship between age and *T. gondii* seropositivity has been inconsistently reported. Some earlier studies indicated that age significantly impacts seroprevalence [45]. Likewise, age was identified as the main factor associated with *T. gondii* infection [46]. A study conducted in an endemic region of Romania revealed an age-related increase in *T. gondii* seroprevalence, with lower rates observed among younger women, and this association is likely due to longer exposure to risk factors over time, along with the fact that infected individuals remain positive for IgG antibodies for years, as *T. gondii* infection is generally considered lifelong [47, 48]. In our findings, age group (OR: 1.23, 95% CI: 0.42–3.84, *p* = 0.81), untreated drinking water (OR: 0.88, 95% CI: 0.33–2.13, *p* = 0.83), and raw meat consumption (OR: 3.29, 95% CI: 0.17–199.90, *p* = 0.56) were associated with higher odds of infection, although none of these associations reached statistical significance. Education level, miscarriage history, and awareness of toxoplasmosis did not significantly affect infection status. This result aligns with findings from a previous study, which suggested that environmental exposure to oocysts may play a more crucial role [49]. Environmental factors have also been widely discussed [50]. For example, soil contact was found to increase the likelihood of seropositivity significantly (OR = 0.482, *p* = 0.015) [12]. Similarly, household gardening was identified as a significant risk factor (*p* = 0.01) [51]. Additionally, living in a house with a garden was associated with a greater risk of *T. gondii* infection (OR = 2.5, 95% CI: 1.21–5.02) [52]. Additionally, a study from midwestern Nepal identified several key risk factors for *T. gondii* infection in sheep and goats, including altitude, animal age, host type, herd size, rearing system, and the presence of cats. These factors may contribute to the risk of zoonotic transmission to humans, especially among individuals in close contact with infected animals [53].

Regarding awareness, the majority of participants in our study lacked knowledge about *T. gondii*, which aligns with studies from Tanzania, where 90% of women reported having never heard of toxoplasmosis [12]. Our findings, which are consistent with those of a study at Western Regional Hospital, Nepal, revealed that none of the participants had knowledge about toxoplasmosis (54). This finding highlights the need for public awareness and health education programs focused on its risks, prevention, and congenital transmission.

## Limitations

This study was aimed at assessing the seroprevalence and associated behavioral factors of *T. gondii* infection among pregnant women in Pokhara Valley, Nepal. While this study provides valuable insights into the epidemiological pattern of toxoplasmosis in this population, several limitations must be acknowledged. In this study, only 91 participants underwent serological testing using both RDT and ELISA. Conducting a larger-scale, long-term study may enable a more accurate estimation of the seroprevalence and the identification of risk factors within the Pokhara municipality. This study was confined to the Pokhara Valley and may not reflect the prevalence or behavioral risk factors for toxoplasmosis in other regions of Nepal, especially rural or high-altitude areas with different environmental and sociocultural conditions. Future studies with larger, randomized samples and longitudinal designs are recommended to validate these findings and explore the temporal relationships between risk factors and infection. Furthermore, this study did not include questions about the specific types of meat consumed. In the future, comparing the culture and current practices related to raw meat consumption across multiple regions in Nepal will be necessary to clarify the detailed risk of *Toxoplasma* infection.

## Conclusions

A total of 91 samples were tested using RDT, revealing a 19.78% IgG seroprevalence with no IgM antibodies detected. In contrast, ELISA demonstrated a higher IgG seroprevalence of 38.46%, also without IgM detection, indicating past exposure to *T. gondii*. Although no statistically significant associations were found for most risk factors, cat ownership was significantly associated with seropositivity, which is consistent with the known transmission pathway of the parasite. Additionally, the assessment revealed suboptimal baseline knowledge about toxoplasmosis among participants, underscoring deficiencies in prenatal education. These findings highlight the need for improved awareness, routine screening, and targeted preventive measures to protect maternal and fetal health from the risks of toxoplasmosis in Nepal.

## Data Availability

The datasets generated and analyzed during the current study are not publicly available due to the inclusion of personally provided data. However, they are available from the corresponding author upon reasonable request with appropriate justification.

## Abbreviations

AUC: Area under the curve
CI: Confidence interval
ELISA: Enzyme-linked immunosorbent assay
GMTH: Gandaki Medical Teaching Hospital
OD: Optical density
OR: Odds ratio
RDT: Rapid diagnostic test
ROC: Receiver operating curve

## References

[1] Liu Q, Das Singla L, Zhou H. Vaccines against *Toxoplasma gondii*: status, challenges and future directions. Hum Vaccin Immunother. 2012;8(9):1305–8. 22906945 10.4161/hv.21006 PMC3579912

[2] Gajadhar AA, Lalonde LF, Al-Adhami B, Singh BB, Lobanov V. Foodborne apicomplexan protozoa: Coccidia. Coccidia. In: Foodborne Parasites in the Food Supply Web: Occurrence and Control. 2015.

[3] Attias M, Teixeira DE, Benchimol M, Vommaro RC, Crepaldi PH, De Souza W. The life-cycle of Toxoplasma gondii reviewed using animations. Parasites and Vectors [Internet]. 2020;13(1):1–13. Available from: 10.1186/s13071-020-04445-z 10.1186/s13071-020-04445-z PMC7686686 33228743

[4] Ali S, Amjad Z, Khan TM, Maalik A, Iftikhar A, Khan I, et al. Occurrence of *Toxoplasma gondii* antibodies and associated risk factors in women in selected districts of Punjab province, Pakistan. Parasitology. 2020;147(10):1133–9. 32517832 10.1017/S0031182020000967 PMC10317724

[5] Eroglu S, Asgin N. Awareness, knowledge and risk factors of *Toxoplasma gondii* infection among pregnant women in the Western Black Sea region of Turkey. J Obstet Gynaecol (Lahore). 2021;41(5):714–20. 10.1080/01443615.2020.1789954. 10.1080/01443615.2020.1789954 33045851

[6] Flegr J, Prandota J, Sovičková M, Israili ZH. Toxoplasmosis - a global threat. Correlation of latent toxoplasmosis with specific disease burden in a set of 88 countries. PLoS ONE. 2014. 10.1371/journal.pone.0090203. 24662942 10.1371/journal.pone.0090203 PMC3963851

[7] Saadatnia G, Golkar M. A review on human toxoplasmosis. Scand J Infect Dis. 2012. 10.3109/00365548.2012.693197. 22831461 10.3109/00365548.2012.693197

[8] Raissi V, Taghipour A, Navi Z, Etemadi S, Sohrabi Z, Sohrabi N, et al. Seroprevalence of *Toxoplasma gondii* and *Toxocara* spp. infections among pregnant women with and without previous abortions in the west of Iran. J Obstet Gynaecol Res. 2020;46(3):382–8. 31953906 10.1111/jog.14184

[9] Chaudhry, et al. Motherisk update: Toxoplasmosis and pregnancy. Can Fam Physician. 2014;60(April):334–6. 24733322 PMC4046541

[10] Asiva Noor Rachmayani. No 主観的健康感を中心とした在宅高齢者における 健康関連指標に関する共分散構造分析Title. 2015;6.

[11] Mandour AM, Mounib MEM, Eldeek HEM, Ahmad AAR, Kader ARMMA. Prevalence of congenital toxoplasmosis in pregnant women with complicated pregnancy outcomes in Assiut Governorate, Egypt. J Adv Parasitol. 2017;4(1):1–8.

[12] Paul E, Kiwelu I, Mmbaga B, Nazareth R, Sabuni E, Maro A, et al. Toxoplasma gondii seroprevalence among pregnant women attending antenatal clinic in Northern Tanzania. Trop Med Health. 2018;46(1):1–8. 30479556 10.1186/s41182-018-0122-9 PMC6245905

[13] Al-Adhroey AH, Mehrass AAKO, Al-Shammakh AA, Ali AD, Akabat MYM, Al-Mekhlafi HM. The prevalence of T. gondii infection among pregnant women varies. BMC Infect Dis. 2019;19(1):1–9. 31888517 10.1186/s12879-019-4718-4 PMC6937662

[14] Blackburn D, Mba N, Nwachukwu W, Zhou H, Hill A, Abbott A, et al. Seroprevalence and risk factors for *Toxoplasma gondii* infection in women of reproductive age in Nigeria in 2018. Am J Trop Med Hyg. 2024;111(5):1005–14. 39255809 10.4269/ajtmh.24-0107 PMC11542513

[15] Rai SK, Sharma A, Shrestha RK, Pradhan P. First case of congenital toxoplasmosis from Nepal. Nepal Med Coll J. 2011;13(1):64–6. 21991707

[16] Rai SK, Kubo T, Yano K, Shibata H, Sumi K, Matsuoka A, et al. Seroepidemiological study of Toxoplasma infection in Central and Western Regions in Nepal. Southeast Asian J Trop Med Public Health. 1996Sep;27(3):548–53. 9185267

[17] McCauley D, Stout V, Gairhe KP, Sadaula A, Dubovi E, Subedi S, et al. Serologic survey of selected pathogens in free-ranging bengal tigers (Panthera tigris tigris) in Nepal. J Wildl Dis. 2021;57(2). 10.7589/JWD-D-20-00046 33822151

[18] Rai SK, Matsumura T, Ono K, Abe A, Hirai K, Rai G, et al. High toxoplasma seroprevalence associated with meat eating habits of locals in Nepal. Asia Pac J Public Health. 1999. 10.1177/101053959901100207. 11195164 10.1177/101053959901100207

[19] Devleesschauwer B, Pruvot M, Joshi DD, De Craeye S, Jennes M, Ale A, et al. Seroprevalence of zoonotic parasites in pigs slaughtered in the Kathmandu valley of Nepal. Vector-Borne and Zoonotic Diseases. 2013. 10.1089/vbz.2013.1313. 24107212 10.1089/vbz.2013.1313

[20] Adhikari P, Pahari R, Joshi SR, Acharya S, Pant S. An unusual presentation of liver abscess secondary to toxoplasmosis in Nepal. Int J Infect Dis. 2022. 10.1016/j.ijid.2021.12.173.36566774

[21] Bhandari S, Kc S, Devkota L, Khadka S, Rai G, Bastola A, et al. Seroprevalence toxoplasma infection among HIV/AIDS patients in Kathmandu, Nepal. Nepal Med Coll J. 2021;23(1):72–5.

[22] Subedi S, Sharma B, Singh S, Bindari YR. Sero-prevalence of Toxoplasma gondii in sheep in different geographical regions of Nepal. Vet Anim Sci [Internet]. 2018;5(January):7–9. Available from: 10.1016/j.vas.2018.01.001 10.1016/j.vas.2018.01.001 PMC7386727 32734039

[23] DoHS. Annual Health Report 2078/2079.

[24] Subedi D, Thakur S, Gautam A, Poudel M, Jyoti S, Devkota A, et al. Milk and meat safety in Nepal: addressing challenges and exploring solutions. Sci One Heal [Internet]. 2025;4(June):100116. Available from: 10.1016/j.soh.2025.100116 10.1016/j.soh.2025.100116 PMC12308024 40740965

[25] Baral R, Gurung K, Bhandari P, Thakuri P, Gurung TW, Sharma SP, et al. Microbiological assessment of broiler chicken meat from different slaughterhouses of Pokhara Valley. Malaysian Anim Husb J. 2023;3(2):98–102.

[26] Bantawa K, Rai K, Subba Limbu D, Khanal H. Food-borne bacterial pathogens in marketed raw meat of Dharan, eastern Nepal. BMC Res Notes. 2018. 10.1186/s13104-018-3722-x. 30157961 10.1186/s13104-018-3722-x PMC6114039

[27] Rai SK, Ono K, Hirai K. Seroprevalence Associated f with Meat Eating Habits o Locals in Nepal. 89–93. 10.1177/101053959901100207 11195164

[28] Khanal S, Kumal A, Shrestha R, Sapkota S, Baral S, K.C S, et al. Microbial quality of chhoyla and kachela: traditional Newari meat products. J Food Sci Technol Nepal. 2020;12(12):9–13.

[29] Ducrocq J, Simon A, Lemire M, De Serres G, Lévesque B. Exposure to *Toxoplasma gondii* through consumption of raw or undercooked meat: a systematic review and meta-analysis. Vector-Borne Zoonotic Diseases. 2021;21(1):40–9. 33202167 10.1089/vbz.2020.2639

[30] Kit HE. Anti-Toxoplasma gondii materials supplied limitations. 2019;(February).

[31] Landis JR, Koch GG. Landis amd Koch1977_agreement of categorical data. Biometrics. 1977;33(1):159–74. 843571

[32] World Health Organization. Basic epidemiology. 2nd ed. Geneva: World Health Organization; 2006.

[33] Singh B, Debrah LB, Acheampong G, Debrah AY. Seroprevalence and Risk Factors of *Toxoplasma gondii* infection among pregnant women in Kumasi: a cross-sectional study at a district-level hospital, Ghana. Infect Dis Obstet Gynecol. 2021;2021. 10.1155/2021/6670219 PMC8041552 33883871

[34] Rishikeshav Acharya M. Study of Toxoplasma gondii, Rubella, CMV and HSV Antibodies among Pregnant Women in Pokhara, Nepal. IOSR J Dent Med Sci e-ISSN [Internet]. 2020;19(January):42–7. Available from: www.iosrjournals.org

[35] Bassiouny HK, Soliman NK, El Tawab S, Eassa SM, Eissa A. Sero-prevalence and risk factors associated with Toxoplasma gondii infection among pregnant women in Alexandria. Egypt Int J Reprod Contracept Obstet Gynecol. 2016;5(12):4220–7. 10.18203/2320-1770.ijrcog20164318.

[36] Hassan JS, Ghazi Hf, Ahmed AARH. Evaluation of rapid chromatographic immunoassay with latex agglutination test and (ELISA) for diagnosis of human toxoplasmosis. J Fac Med Baghdad. 2011;52(4):468–70.

[37] Bannu D, Kp KP. Prevalence of Toxoplasma gondii antibodies among pregnant women in Prevalence of Toxoplasma gondii antibodies among pregnant women in District Bannu , Khyber Pakhtunkhwa ( KP ), Pakistan. 2018;(October 2020).

[38] Lamichhane B, Pudasaini B, Upadhyay B, Sharma M, Khanal SP. Prevalence of serum antibodies to TORCH infections among the women of child bearing age visiting National Public Health Laboratory, Teku. Nepal J Sci Technol. 2015;15(2):85–90.

[39] Sah RP, Talukder MH, Rahman AKMA. Serodiagnosis of toxoplasmosis using lateral flow chromatographic immunoassay among animals and humans in Sunsari district of Nepal. Nepalese Vet J. 2018;35:98–109.

[40] Chandrasena N, Herath R, Rupasinghe N, Samarasinghe B, Samaranayake H, Kastuririratne A, et al. Toxoplasmosis awareness, seroprevalence and risk behavior among pregnant women in the Gampaha district, Sri Lanka. Pathog Glob Health. 2016;110(2):62–7. 27092763 10.1080/20477724.2016.1173325 PMC4894262

[41] Agordzo SK, Badu K, Addo MG, Owusu CK, Mutala AH, Tweneboah A, et al. Seroprevalence, risk factors and impact of *Toxoplasma gondii* infection on haematological parameters in the Ashanti region of Ghana: a cross-sectional study. AAS Open Res. 2020;2:166. 32734139 10.12688/aasopenres.13022.1 PMC7369427

[42] Adhikari RB, Dhakal MA, Ale PB, Regmi GR, Ghimire TR. Survey on the prevalence of intestinal parasites in domestic cats (*Felis catus* Linnaeus, 1758) in central Nepal. Vet Med Sci. 2023;9(2):559–71. 36346533 10.1002/vms3.999 PMC10029910

[43] Sakikawa M, Noda S, Hanaoka M, Nakayama H, Hojo S, Kakinoki S, et al. Anti-Toxoplasma antibody prevalence, primary infection rate, and risk factors in a study of toxoplasmosis in 4,466 pregnant women in Japan. Clin Vaccine Immunol. 2012;19(3):365–7. 22205659 10.1128/CVI.05486-11 PMC3294603

[44] Soltani S, Ghaffari AD, Kahvaz MS, Sabaghan M, Pashmforosh M, Foroutan M. Detection of Anti-*Toxoplasma gondii* IgG and IgM antibodies and associated risk factors during pregnancy in Southwest Iran. Infect Dis Obstet Gynecol. 2021;2021. 10.1155/2021/5547667 PMC8175175 34135564

[45] Laboudi M, Taghy Z, Duieb O, Peyron F, Sadak A. *Toxoplasma gondii* seroprevalence among pregnant women in Rabat, Morocco. Trop Med Health. 2021. 10.1186/s41182-021-00311-5. 33685529 10.1186/s41182-021-00311-5 PMC7941977

[46] Rostami A, Seyyedtabaei SJ, Aghamolaie S, Behniafar H, Lasjerdi Z, Abdolrasouli A, et al. Seroprevalence and risk factors associated with toxoplasma gondii infection among rural communities in northern Iran. Rev Inst Med Trop Sao Paulo. 2016;58(12):4220–7. 10.1590/S1678-9946201658070 PMC5048641 27680175

[47] Mihu AG, Balta C, Marti DT, Paduraru AA, Lupu MA, Olariu TR. Seroprevalence of *Toxoplasma gondii* infection among women of childbearing age in an endemic region of Romania, 2016–2018. Parasite. 2020;27:6–9. 33198884 10.1051/parasite/2020057 PMC7669453

[48] dan Bree, Dara; levy. 乳鼠心肌提取 HHS Public Access. Physiol Behav. 2019;176(3):139–48.

[49] Mosawi SH, Zarghona Z, Dalimi A, Jokelainen P, Safa AH, Mohammadi MR, et al. Particularly neglected in countries with other challenges: High Toxoplasma gondii seroprevalence in pregnant women in Kabul, Afghanistan, while a low proportion know about the parasite. PLoS One [Internet]. 2019;14(10):1–9. Available from: 10.1371/journal.pone.0223585 10.1371/journal.pone.0223585 PMC6786618 31600338

[50] Siddiqui N, Shujatullah F, Khan HM, Rabbani T, Khan PA. IgG avidity antibodies against *toxoplasma gondii* in high risk females of reproductive age group in India. Korean J Parasitol. 2014;52(5):487–91. 25352696 10.3347/kjp.2014.52.5.487 PMC4210730

[51] Iddawela D, Vithana SMP, Ratnayake C. Seroprevalence of toxoplasmosis and risk factors of *Toxoplasma gondii* infection among pregnant women in Sri Lanka: a cross sectional study. BMC Public Health. 2017;17(1):1–6. 29202747 10.1186/s12889-017-4941-0 PMC5716377

[52] Marković-Denić L, Stopić M, Bobić B, Nikolić V, Djilas I, Srzentić SJ, et al. Factors associated with *Toxoplasma gondii* seroprevalence in pregnant women: a cross-sectional study in Belgrade, Serbia. Pathogens. 2023. 10.3390/pathogens12101240. 37887756 10.3390/pathogens12101240 PMC10610184

[53] Sharma BB, Dahal A, Bhattarai RK, Singh P, Shrestha P, Adhikari A, et al. Seroprevalence and associated risk factors of. 2025.

[54] Jaiswal S, Pokhrel T, Sharma S. Seropositivity Rates of Toxoplasmosis and Syphilis in Pregnant Women Visiting Western Regional Hospital, Nepal. … J Heal … [Internet]. 2014;(August). Available from: http://www.researchgate.net/profile/Suresh_Jaiswal/publication/269694364_International_Journal_of_Health_Sciences_and_Research_Seropositivity_Rates_of_Toxoplasmosis_and_Syphilis_in_Pregnant_Women_Visiting_Western_Regional_Hospital_Nepal/links/549272c00cf2

